# A Stalled Revolution? Misoprostol and the Pharmaceuticalization of Reproductive Health in Francophone Africa

**DOI:** 10.3389/fsoc.2021.590556

**Published:** 2021-04-12

**Authors:** Siri Suh

**Affiliations:** Brandeis University, Waltham, MA, United States

**Keywords:** misoprostol, reproductive health, abortion, Francophone Africa, pharmaceuticalization, population control

## Abstract

Misoprostol entered the global market under the name Cytotec in the mid-1980s for the treatment of gastric ulcers. Decades of research have since demonstrated the safety and effectiveness of off-label use of misoprostol as a uterotonic in pregnant women to prevent and treat post-partum hemorrhage, treat incomplete abortion, or terminate first-trimester pregnancy. Global health experts emphasize misoprostol’s potential to revolutionize access to reproductive health care in developing countries. Misoprostol does not require refrigeration, can be self-administered or with the aid of a non-physician, and is relatively inexpensive. It holds particular promise for improving reproductive health in sub-Saharan Africa, where most global maternal mortality related to post-partum hemorrhage and unsafe abortion occurs. Although misoprostol has been widely recognized as an essential obstetric medication, its application remains highly contested precisely because it disrupts medical and legal authority over pregnancy, delivery, and abortion. I draw on fieldwork in Francophone Africa to explore how global health organizations have negotiated misoprostol’s abortifacient qualities in their reproductive health work. I focus on this region not only because it has some of the world’s highest rates of maternal mortality, but also fertility, thereby situating misoprostol in a longer history of family planning programs in a region designated as a zone of overpopulation since the 1980s. Findings suggest that stakeholders adopt strategies that directly address safe abortion on the one hand, and integrate misoprostol into existing clinical protocols and pharmaceutical supply systems for legal obstetric indications on the other. Although misoprostol has generated important partnerships among regional stakeholders invested in reducing fertility and maternal mortality, the stigma of abortion stalls its integration into routine obstetric care and availability to the public. I demonstrate the promises and pitfalls of pharmaceuticalizing reproductive health: despite the availability of misoprostol in some health facilities and pharmacies, low-income and rural women continue to lack access not only to the drug, but to quality reproductive health care more generally.

## Introduction

Misoprostol offers a particularly vivid example of how pharmaceuticals inhabit multiple “social lives” or “biographies” throughout various stages of their production, marketing, distribution, prescription, and consumption (Geest et al., 1996; Whyte et al., 2002). In the late 1980s, medical professionals in Brazil began reporting that women who used misoprostol to terminate pregnancy experienced less severe complications of incomplete abortion (Coêlho et al., 1994; Löwy and Dias Villela Corrêa, 2020). Produced under the brand name Cytotec by pharmaceutical giant Pfizer, this drug had been available on the global market since 1985 for the treatment of gastric ulcers. In pregnant women, misoprostol acts as a uterotonic, causing the cervix to soften and the uterus to contract. In addition to terminating pregnancy, misoprostol may also be used to induce labor, prevent and treat post-partum hemorrhage (PPH), and treat complications of incomplete abortion (also known as post-abortion care or PAC). While off-label utilization of misoprostol is challenging to measure, in 2007 an estimated total of 129,300 mcg of misoprostol were sold in pharmacies and hospitals worldwide. Off-label use of misoprostol is thought to be highest in Asia, where the drug has been approved for managing PPH and medication abortion (MA) where legal, and where the cost of the drug is the lowest (Fernandez et al., 2009). While misoprostol is sold in brick and mortar health facilities and pharmacies, it may also be purchased from informal drug vendors, and where available, the Internet. For example, since launching in 2018, the online organization Aid Access has distributed at least 600 medication abortion kits including misoprostol to women in the United States (Lussenhop, 2018).

Although World Health Organization (WHO) listed mifepristone and misoprostol used together as a safe form of early MA in 2005, clinical research has shown that misoprostol alone can effectively terminate first-trimester pregnancy (von Hertzen et al., 2007). WHO placed misoprostol on its List of Essential Medications (LEM) for labor induction in 2005, for PAC in 2010, for preventing PPH in 2011, and for treating PPH in 2015 (WHO, 2019a). More recently, in 2018, WHO listed misoprostol alone as an alternative to the recommended combination regimen for MA (WHO, 2019b). In response to increasing access to MA worldwide, WHO shifted its abortion classification system. While epidemiologists had previously differentiated between safe and unsafe abortion in terms of the legality of the procedure (safe abortions are legal, unsafe abortions are illegal), abortions are now classified along a spectrum from safe, less safe, to least safe (Ganatra et al., 2017).

In both popular media and public health literature, misoprostol has been described as a “revolution” in reproductive health care because of its potential to reduce mortality and morbidity related to unsafe abortion and PPH in countries with restrictive abortion laws or with under-resourced health systems (Kristof, 2010; WLP, 2010; Henderson et al., 2012; Harvey, 2015). Misoprostol is an effective alternative to oxytocin for both the prevention and treatment of PPH (Raghavan, Abbas, and Winikoff, 2012; Sheldon et al., 2012). Furthermore, studies have shown that community-based health workers such as traditional birth attendants (TBAs) can safely administer misoprostol to prevent PPH (Prata et al., 2012; Smith et al., 2013; Bell et al., 2014). The drug is equally as effective as the manual vacuum aspiration (MVA) syringe in administering PAC (Dao et al., 2007; Ibiyemi et al., 2019). Generic brands of misoprostol are sold in smaller quantities and thus available with or without prescription at a lower price than the brand name Cytotec (sold in quantities of at least 14 tablets), in brick and mortar pharmacies and in informal drug markets. Additionally, misoprostol does not require refrigeration and can be used safely in low-resource settings.

Misoprostol holds particular promise for pharmaceutically revolutionizing women’s access to quality reproductive care in sub-Saharan Africa (SSA), where the burden of abortion-related mortality is the highest in the world (nearly 62% of global abortion deaths occurred in Africa in 2008 [WHO, 2011a]), where hemorrhage accounts for 25% of maternal death (Say et al., 2014), and where there are only 2.2 health workers per 1,000 population (WHO, 2016). Indeed, the availability of misoprostol for safe abortion, PAC, and PPH could avert more maternal deaths than other large-scale interventions in developing countries (Prata et al., 2009).

Critical scholars of global health are more skeptical about the extent to which medication alone can lead to a revolution in access to health care. More specifically, they have cautioned against reducing solutions to complex public health problems like maternal mortality to “magic bullets”: cost-effective drugs or interventions that promise to save the most lives at the lowest cost, but with inadequate attention to the social and economic context in which these technologies are applied (or not) (Farmer et al., 2013; Adams, 2016). Magic bullet approaches have been incentivized within a broader landscape of neoliberal health reform since the late 1970s, in which the competitive distribution of health goods and services through the market, rather than the government, purportedly leads to greater standardization, accountability, access, and quality (Erikson, 2012; Basilico et al., 2013; Chorev, 2013). In developing countries, structural economic reform, led by the International Monetary Fund (IMF) and the World Bank, has decreased government support for health care, leaving private organizations to fill in service delivery gaps (Pfeiffer and Chapman, 2010; Packard, 2016; Pfeiffer, 2019). Within a context of economic scarcity, aid donors and governments prioritized the implementation of practical, technologically driven approaches whose effectiveness can be statistically measured, ideally through randomized controlled trials (Adams, 2013; Adams, 2016). For example, starting in the 1980s, the World Bank supported a collection of primary health care interventions for children—growth monitoring, oral rehydration therapy, breast feeding, and immunizations—known as GOBI (Basilico et al., 2013; Packard, 2016).

The desire for technological solutions and measurable results has led to what critical scholars of global health have termed the “pharmaceuticalization” of public health, a process through which medication comes to stand in for health infrastructure and human resources that constitute public health systems (Biehl, 2006; Biehl, 2007; Bell and Figert, 2012). For example, studies in Brazil and Mozambique have shown how HIV/AIDS programs that prioritize the availability of anti-retroviral therapy (ART) overlook, and in some cases reinforce, inequalities in basic health needs such as nutrition, housing, and clean water that increase people’s vulnerability to HIV infection (Biehl, 2006; Biehl, 2007; Kalofonos, 2010). In Tanzania, mass administration of deworming medication took precedence over environmental interventions to reduce the burden of parasitically transmitted diseases such as onchocerciasis and trachoma (Samsky, 2015). Even in wealthy countries like the United States, stratified access to life-saving drugs and technologies reinforces racial and class inequalities in health outcomes (Clarke et al., 2003; Phelan et al., 2010). While medication may effectively treat disease, pharmaceutical approaches do not adequately address underlying inequalities that render some people at greater risk of disease and limit their access to preventive and curative care. From this perspective, misoprostol alone may not be enough to reduce maternal mortality in SSA.

Framing misoprostol as a revolution in reproductive health care further belies the complex geopolitical and professional processes—a transnational “regime of values” (Geest et al., 1996)”—involved in approving and registering drugs, determining who can administer them and how, and in making them available (and affordable) to relevant populations. Despite misoprostol’s potential to reduce maternal death, its off-label obstetric indications, and in particular its capacity to terminate pregnancy, have rendered it a “pharmaceutical outlaw” (MacDonald, 2020) in a global arena of reproductive health that remains reluctant to accept abortion as a legitimate part of obstetric care (Suh, 2018). For example, US policies like the 1973 Helms Amendment and the 1984 Mexico City Policy (also known as the Global Gag Rule [GGR]) prohibit US development assistance from being used to procure abortifacient technologies or to support abortion-related activities in developing countries. As recently as May 2020, in a letter to the UN Secretary General, US Agency for International Development (USAID) threatened to withdraw funding from the UN Humanitarian Response to the global Covid-19 pandemic because of its alleged “promotion” of abortion through “the widespread distribution of abortion-inducing drugs and abortion supplies” (USAID, 2020). Additionally, faith-based, anti-abortion organizations such as Maternal Life International have expressed opposition to WHO’s inclusion of misoprostol for preventing PPH on its LEM, arguing that it promotes a lower standard of obstetric care outside the hospital for pregnant women in developing countries (Adams, 2020).

Misoprostol’s relatively recent “biography” as an abortifacient is connected to a longer history of (neo)colonial population governance in SSA. In this article, I explore politics and practices related to misoprostol in the Francophone region of SSA, which includes countries in West and Central Africa. I focus on this region not only because it has some of the world’s highest rates of maternal mortality, but also the highest rates of fertility and lowest rates of modern contraceptive use. Since at least the 1980s, family planning has factored prominently into structural adjustment policies designed to increase economic growth throughout the region under the guidance of the IMF and the World Bank (Hartmann, 1995; Robinson, 2015). By the turn of the millennium, Francophone Africa was designated as a climate change “hot spot” (Mutunga et al., 2012) due to population growth. By focusing on this region, I locate the misoprostol revolution in a longer geopolitical history of magic bullet approaches to address population problems in this region.

In this article, I situate misoprostol, a highly flexible reproductive health drug with multiple obstetric indications, in a fractured landscape of reproductive governance (Morgan and Roberts, 2012) that includes population control, maternal mortality reduction, safe abortion, and PAC. I draw on in-depth interviews conducted in 2019 with individuals from national and international non-governmental organizations (NGOs) and philanthropic agencies currently engaged in reproductive health research, advocacy, and service delivery in the region, and a review of national, regional, and global literature on misoprostol. My findings show that these organizations pragmatically engage in approaches that, on the one hand, openly signal their support for safe abortion through training and advocacy, and quietly support the approval of misoprostol for legitimate obstetric indications by national pharmaceutical regulatory agencies and its integration into national LEM and private and public pharmaceutical supply systems, on the other. For advocates of safe abortion, both approaches are necessary in a region where abortion laws remain highly restrictive, USAID remains an influential donor of reproductive health aid, and health officials and medical workers face immense pressure to meet national and global demographic targets to reduce fertility and maternal mortality.

By exploring how these actors integrate misoprostol into health policy and practice, I offer insight into the benefits, challenges, and pitfalls of pharmaceuticalizing reproductive health. I argue that access to misoprostol cannot simply be boiled down to its availability in pharmacies, or its affordability on the market. The narrative of revolution, while hopeful about the drug’s potential to reduce maternal mortality, insufficiently captures the complex transnational politics that shape how and where it is available and used (or not), and by whom. Attention to global, national, and regional practices, strategies, and discourses related to misoprostol reveals how access to pharmaceuticals falls short of reproductive justice in a region significantly influenced by gendered, racialized, and classed geopolitics of reproduction.

## Methods

This study was accomplished through ethnographic research conducted between July and August 2019 in Dakar, Senegal. My research on misoprostol stems from a longer professional and scholarly engagement with reproductive health, and in particular PAC, in Senegal. During the mid-2000s, I worked with Management Sciences for Health (an international health NGO) on PAC in five of USAID’s regions of intervention in Senegal. Between 2009 and 2011, I conducted research on PAC in Senegal, and since then have remained connected with colleagues in the Ministry of Health (MOH) and national and international NGOs involved in reproductive health research and programming.

First, I conducted in-depth, semi-structured interviews with 17 individuals involved in reproductive health care advocacy, research, and programming in Francophone Africa. Interviews with participants in Senegal occurred in person, while others took place over Skype with participants based throughout Francophone Africa, Europe, and the US. At the time of fieldwork, research participants worked with philanthropic agencies and NGOs. While some participants had worked in the past with national MOH or as health workers in government health facilities, I did not interview current MOH officials or government health workers as part of this study. Participants represented a variety of nationalities including Senegalese, Ivorian, Burkinabé, American, British, and Dutch. Although I purposefully selected some participants due to their organizational positions, others were referred to me through snowball sampling. This personalized approach, and my previous research experience in PAC in Senegal, facilitated sensitive conversations about misoprostol and abortion. The names of individuals and organizations have been anonymized to protect confidentiality. Permission for this project was obtained from Brandeis University and the Ministère de l'Enseignement Supérieure, de la Recherche, et de l’Innovation in Senegal.

Second, I conducted literature reviews on misoprostol and MA. Sources include clinical research on misoprostol’s safety and efficacy in obstetric care; technical guidelines on abortion care issued by WHO; sociological and anthropological literature on women’s and health workers’ experiences with misoprostol in SSA; and public health literature on availability and use of misoprostol in public and private health care sectors. Also included in my analysis are data from reports on MA published by international NGOs and donor agencies. Some of these reports describe the proceedings of stakeholder meetings, while others outline national and regional strategies for expanding access to MA in Francophone Africa. In light of the sensitive nature of abortion in this context, I do not reveal the identities of individuals or organizations involved in the publication of these reports, unless the report is available to the public online.

Drawing on multiple sources of data, I identify various institutions, professions, industries, communities, and technologies that make up the complex and rapidly evolving “pharmaceutical regime” (Biehl, 2006: p. 207) of misoprostol in Francophone Africa. This approach offers insight into how misoprostol is simultaneously entangled with and isolated from global, regional, and national goals, discourses, and policies related to maternal and reproductive health. While tracing how misoprostol activities are unfolding on the ground, this methodological approach allows us to see how access remains limited for some populations. I draw on the mapping exercises and situational analyses of MA in NGO reports to supplement study participants’ explanations of misoprostol marketing and registration. I compare participants’ perceptions and descriptions of misoprostol strategies and practices with national and regional goals established for MA during global and regional stakeholder meetings. I juxtapose participants’ perceptions of misoprostol practices among health workers and women with findings from ethnographic research on misoprostol in SSA. Where possible, I draw on national surveys to estimate the availability of misoprostol in public health facilities.

## Theoretical Significance

Science and technology studies and critical studies of global health offer useful theoretical tools to understand the production, marketization, distribution, prescription, and availability of pharmaceuticals across a variety of contexts. In this section, I draw on these tools to illuminate the gap between misoprostol’s promise as an obstetric pharmaceutical in transnational arenas of maternal and reproductive health policy making and the practical realities of misoprostol on the ground. Pharmaceuticals are not inherently safe or effective, but acquire meaning through the social, economic, and political dimensions of their utilization (Timmermans and Berg, 2003; Casper and Morrison, 2010). As pharmaceuticals circulate through various “lives” or “biographies” related to experimentation, production, or consumption, they are embedded in shifting “regimes of values,” or ideas about efficacy, disease, treatment, and patient populations (Geest et al., 1996; Whyte et al., 2002). Pharmaceuticals are never finished products, but are engaged in complex “human and non-human processes and practices (p. 119)” through which they are continually “made and remade (p. 118)” (Hardon and Sanabria, 2017).

Professional and lay debates about what makes a particular drug or technology “the right tool for the job” (Clarke and Fujimura, 1992), and for whom the drug is appropriate, reveal inequalities along the lines of gender, race, class, and nationality with respect to who can and should use pharmaceutical products and for what purpose. In the United States, for example, opposition to the Human Papillomavirus (HPV) vaccine on the grounds that it would encourage promiscuity, especially among young girls, delayed its integration into pediatric care (Mamo and Epstein, 2014). Some scholars have attributed the delay in expanding access to ART in SSA to racist perceptions of Africans’ inability to follow drug regimens, and the drug resistance that would surely arise from non-compliance (Nauta, 2010; Crane, 2011).

The “job” that pharmaceuticals are designed to accomplish, or their “biography” at a particular moment in time, may shift in response to social, professional, economic or political factors. Pharmaceutical companies have encouraged off-label prescription of drugs, despite a lack of clinical evidence, to maximize profits through expanded patient populations. Examples include drugs for sexual disorders, social anxiety, Attention Deficit Hyperactivity Disorder (ADHD), depression, and other mental illnesses (Abraham, 2010). Off-label prescription of drugs is neither uncommon nor new: a 1998 study found that nearly 39% of drug prescriptions in pediatric wards of European hospitals were off-label (Conroy et al., 2000). Some off-label “jobs” may be deemed too controversial by pharmaceutical companies to promote in clinical practice. Despite the integration of misoprostol into national and global guidelines for obstetric care, Pfizer has not applied for a license to cover the reproductive health indications of Cytotec, a drug that is registered for treatment of ulcers in at least 80 countries (Weeks et al., 2005). Nevertheless, Pfizer likely profits from off-label utilization of misoprostol for abortion (Morgan, 2019).

In the global South, pharmaceuticals are “reinscribed (p. 120)” with meaning related to safety, efficacy, and patient populations not only in clinical trials, consumer markets, and health care settings (Hardon and Sanabria, 2017), but also in health policy arenas according to “shifting global priorities and funding arrangements (p. 150)” (Hardon and Dilger, 2011). This terrain is composed not only of UN agencies, international NGOs, and bilateral donors, but increasingly “philanthrocapitalist” organizations like the Gates Foundation (Birn, 2014) and pharmaceutical companies. In Brazil, for example, anthropologist João Biehl describes the role of the World Bank and global pharmaceutical companies in a “pharmaceutical regime” that made ART universally available to the population in 1996 (Biehl, 2006).

Misoprostol’s potential to improve reproductive health has been framed as revolutionary precisely because beyond its capacity as an abortifacient, it is the “right tool” for multiple obstetric “jobs,” including labor induction, preventing and treating PPH, and PAC. In this sense, misoprostol is anchored in multiple “regimes of value” related to global efforts to reduce maternal mortality. Although the 1987 Safe Motherhood Initiative called on donors and governments to invest in maternal health care comprehensively as a matter of social justice (Smith and Shiffman, 2016), its twenty-first century iteration—Women Deliver—has demanded investment in specific interventions that have demonstrated, through statistical evidence, cost-effectiveness in reducing maternal death (Storeng and Behague, 2014; Storeng and Behague, 2017). Misoprostol is the ideal “silver bullet (p. 272)” for maternal health because its cost-effectiveness in preventing and treating PPH and administering PAC has been demonstrated statistically in randomized controlled trials (Storeng and Béhague, 2014).

At the same time, despite its proven safety record and integration into national and global LEM, misoprostol’s capacity to terminate pregnancy continues to threaten its legitimacy as a reproductive health drug (Starrs and Winikoff, 2012; MacDonald, 2020) and renders it incompatible with US funding mechanisms for family planning aid. Despite USAID support for research and programming on misoprostol for PPH and PAC (Grenier et al., 2013; Barot, 2014), misoprostol cannot be procured with US development assistance under the 1973 Helms Amendment, which prohibits the “performance of abortion as a form of family planning (Barot, 2013: p. 9).” In addition to donations of misoprostol from NGOs and other agencies, national health authorities must purchase the drug from wholesale distributors, at times leading to gaps and inconsistencies in supply (Samnani et al., 2017). In this sense, the anti-abortion funding restrictions of the largest donor of global reproductive health aid (Grollman et al., 2018) may influence supplies of misoprostol in government health facilities.

Even for a legitimate obstetric indication like PPH, misoprostol’s capacity to disrupt medical power over pregnancy, abortion, and delivery continues to generate professional debates over where it should be used, and by whom (MacDonald, 2020). Indeed, misoprostol disrupts conventional knowledge about where maternal health care itself should be administered. Since the 2000s, global maternal health authorities have stressed the importance of skilled birth attendance (births attended by doctors, nurses, or midwives), ideally in well-equipped health facilities, to reduce maternal death (Stanton 2008). The 2015 Sustainable Development Goals have maintained skilled birth attendance as a key indicator of progress toward reducing maternal death (Chou et al., 2015). Yet, even as WHO and other global maternal health stakeholders have promoted skilled birth attendance, other maternal health advocates have critiqued this approach, pointing to evidence that TBAs can use misoprostol in community-based settings to safely and effectively manage PPH (Potts et al., 2006; Potts and Hammerling, 2006).

Some maternal health scientists and organizations have been critical of WHO’s recognition of misoprostol as an alternative medication for preventing and treating PPH in the absence of the gold standard, oxytocin. In 2011, Maternal Life International, a faith-based organization, wrote to WHO to question the evidence base for misoprostol’s inclusion on the LEM for preventing PPH, arguing that it fostered sub-standard, unregulated care outside of health facilities for poor, rural women in SSA (WHO, 2011a; Adams, 2020). Similarly, in 2018, researchers from Newcastle University in the United Kingdom requested that WHO remove misoprostol for the prevention of PPH from its LEM in light of a lack of rigorous statistical evidence supporting community-based administration of the medication (WHO, 2019b). In counter-response, Médecins Sans Frontières (MSF) argued that misoprostol must remain on the LEM to ensure “an alternative for prevention of PPH in resource-poor community and rural settings where injectable oxytocics are not available, or cannot be safely administered” (WHO, 2019c). Additionally, some misoprostol advocates have argued that in some developing countries, it may be financially, ethically, and logistically “impossible” to conduct randomized, placebo-controlled trials that produce rigorous statistical evidence of the medication’s efficacy in managing PPH, and that demands for additional statistical evidence unjustly delay women’s access to safe and effective care (Potts et al., 2010).

Given the recent resurgence of attention to family planning as a global health matter, surprisingly little attention has been directed to misoprostol’s capacity, as an abortifacient, to achieve global targets related to fertility reduction. Compared to modern contraception, abortion plays a limited role in fertility reduction. Yet, the “contraception-abortion paradox (p. 12)” exists in many developing countries, where limited access to or inconsistent use of contraception increases the risk of unwanted pregnancy, which in turn frequently leads to abortion (WHO, 2011b). There is precedent for the deployment of abortion technologies, along with long-acting reversible contraceptives (LARCs), in the achievement of fertility reduction goals in the global South. Starting in the late 1960s, modern contraception was identified as a technical solution to the perceived problem of overpopulation in newly sovereign nations in the global South. Neo-Malthusian predictions of political and economic instability and environmental degradation due to overpopulation catalyzed an era widely known as “population control,” during which developing countries were compelled to establish population policies that articulated fertility reduction goals in exchange for development assistance from bilateral and multilateral donors. Through family planning programs established by international NGOs, USAID “inundated” developing countries with low-cost contraceptives (Murphy, 2012; Takeshita, 2012). These programs have been widely critiqued for coercive practices and inadequate attention to women’s needs, comfort, and safety (Hartmann, 1995; Kuumba, 1999).

Before the MVA syringe became a preferred technology for PAC in the early 1990s (Suh, 2015), it was promoted by USAID as a technology for “menstrual regulation” (a euphemism for abortion) in parts of Asia and Latin America during the early 1970s (Murphy, 2012). After the passage of the 1973 Helms Amendment, USAID delegated MVA research and distribution to NGOs such as International Planned Parenthood Federation (IPPF), Pathfinder International, and Ipas. Even with the Helms Amendment and the GGR in place, MVA had been distributed in over 100 countries by 1993 (Adams, 2018).

At the 1994 UN International Conference on Population and Development (ICPD), the global community rejected the target-oriented population control paradigm in favor of the concept of reproductive health, which called for comprehensive, rights-based approaches to ensuring women’s reproductive well-being. By the early 2000s, however, fertility reduction through voluntary family planning emerged yet again as a key component of global approaches to address the environmental dangers of climate change (Sasser, 2018). Over the next decade, SSA was identified as a region particularly vulnerable to the impact of climate change on food security, water, and health. The Gates Foundation explicitly identified population growth as a cause of “disease burden, environmental degradation, poverty, and conflict” (Bill and Melinda Gates Foundation, 2012). Nearly all of the 26 countries designated as population and climate “hotspots”—areas characterized by “high rates of population growth, high projected declines in agricultural production and low resilience to climate change” (PAI, 2011, p. 2)—in 2011 were in SSA. As the “primary collectors” of food, water, and fuel in SSA, women were more vulnerable to climate change and therefore the ideal target population for contraception to help them “cope” (Mutunga et al., 2012, p. 12).

In SSA, family planning in the form of “birth spacing” has been tightly connected to maternal health through its capacity to avert undesired or unplanned pregnancies (Duclos et al., 2019; Brunson, 2020). In 2012, the global reproductive health community called for revitalized investment in fertility reduction. The Family Planning 2020 Initiative (FP 2020), with over one billion dollars in funding from the Gates Foundation, committed to providing 120 million women with modern contraception by 2020 in 69 of the world’s poorest countries. Over half (58%) of FP 2020 countries are in Africa. In 2012, nine Francophone African countries formed the Ouagadougou Partnership (OP), a regional collective that aimed to add an additional 2.2 million contraceptive users by 2020. Donors and NGOs have worked with OP countries to establish targets for number of users and contraceptive prevalence (Bendix et al., 2019). With financial support from the Gates Foundation, global pharmaceutical companies and NGOs have redesigned and tested LARCs like Depo Provera and Norplant for widespread use in SSA (Hartmann, 2014; Bendix et al., 2019).

Feminist scholars have argued that population, development, and climate interventions like FP 2020 must be situated in a longer history of (neo)colonial strategies to control African fertility (Kuumba, 1999). Some colonial population policies were designed to increase fertility, ensuring a robust colonial labor supply (Knoppers et al., 1990; Hunt, 1999), while others aimed to curtail African fertility to shore up white supremacy (Brown, 1987; West, 1994; Klausen, 2016). More recently, at the 2017 G20 Summit, French President Emmanuel Macron was accused of racism when he framed high fertility rates in SSA as a “civilizational” problem (Wintour, 2018).

Although FP 2020 uses the language of reproductive rights and maternal mortality reduction to frame contraceptive use, it reanimates neo-Malthusian logics of population control that posit fertility in SSA as a threat to the economic, political, and environmental stability— “the abundant life” (Murphy, 2017)—of wealthy countries in the global North (Hartmann, 2014; Bhatia et al., 2019). Such initiatives prioritize a technical solution—contraception—to complex social, political, and economic problems such as food insecurity, land dispossession, and disinvestment in subsistence agriculture (much of which is performed by women in SSA [Rodgers and Akram-Lodhi, 2019]). They disproportionately burden women in SSA for solving the problem of climate change, despite the fact that at 3%, this region has the lowest greenhouse gas emissions in the world (Sy, 2016). They reduce women to mere instruments through which governments can manipulate problematic demographic characteristics—such as SSA’s “youth bulge”—to maximize economic growth (Nyambura, 2018). Put differently, the nexus between population, development, and climate change exemplified by global interventions like FP 2020 reinforce race, gender, and class hierarchies that devalue the reproduction of women of color in the global South.

Through its multiple “lives,” misoprostol is anchored in multiple global regimes of maternal and reproductive health governance. And yet, the qualities that render it a potentially revolutionary medication for achieving maternal mortality reduction simultaneously raise the specter of unregulated abortion and increased participation of TBAs in obstetric care. As an abortifacient, misoprostol’s ties to a neo-Malthusian past and present threaten to destabilize precarious global agreements on achieving fertility reduction targets in a post-ICPD landscape. In the following sections, I explore how reproductive health advocates have negotiated these qualities in their efforts to increase the availability of misoprostol in Francophone Africa.

## “Right at the Precipice”: Regional Prioritization of Safe Abortion

Some study participants are explicitly engaged in advancing access to MA, and safe abortion more generally, in Francophone Africa as part of a broader maternal and reproductive health agenda. Abortion-specific meetings have been held, either as stand-alone events or tied to other global or regional maternal and reproductive health conferences starting in 2016. Some of these events have been organized and held in secrecy because of the politically sensitive nature of abortion. These meetings have been attended by representatives from national and international NGOs, research organizations, bilateral donor agencies, UN agencies, and philanthropic agencies. During these meetings (none of which I attended), safe abortion advocates have discussed trends, progress, and challenges related to MA in the areas of research, legal environment, advocacy, commodities, community engagement, funding priorities, and services. For example, during a January 2019 meeting on safe abortion, almost 40% of participants indicated that their funds were allocated to MA services, and 88% identified Francophone West Africa as a priority region (SAD, 2019a). Safe abortion advocates use these meetings to rethink priorities, conduct situational analyses, coordinate and synergize activities, and articulate next steps.

Catherine, Maxine, and Roberta, who worked for two philanthropic foundations, identified reproductive health more generally as a long-standing goal within their funding portfolios related to “population.” Although these foundations have supported safe abortion advocacy and research among national and international NGOs for several decades, they explained that the 2012 OP served as a catalyst for increased regional interest in safe abortion, with a particular focus on MA. They identified new actors, including NGOs and bilateral donor agencies, that had become involved in family planning, and eventually began to dedicate attention and resources to safe abortion. Catherine believed that despite the OP’s focus on family planning, this initiative had catalyzed a regional approach to MA that was poised to transform the landscape of abortion:

There’s a lot more interest as a region, the conceptualization of the region as a space, thinking of it regionally, like these are lots of small-ish countries so when we think regionally, a lot can be done. Donors think regionally. I think it’s been great and catalytic for safe abortion work. And of course, there’s the MA revolution happening all this time and that has really changed safe abortion work globally, of course. But I think it’s right at the precipice of being extremely fundamentally shifting, especially in Francophone Africa.

Maxine agreed that there had been a “general shift towards West Africa,” but did not attribute it solely to safe abortion. Instead, she located it in a broader recognition within the global reproductive health community that Francophone Africa was underserved compared to other regions in SSA. Some stakeholders have identified the region’s official language as a deterrent among Anglophone donors (SAD, 2019b). Prior to working with her foundation, Maxine had been engaged in reproductive health work with another organization in the region and felt as if “we were begging for support for our programs in West Africa.” While there was some support from a few European bilateral agencies, “it was hard to get other donor support because it looked like there were more opportunities and movement and government support in East Africa, and to some extent, some of the Central African countries.” She linked these geographic foci to a “huge surge” of interests and resources related to HIV/AIDS. She believed that stakeholders had recently turned their attention to Francophone Africa as a place that was “still in need.” For her, “there has been a general growing interest in West Africa around reproductive health, and then, maybe safe abortion is a part of that.”

Study participants who worked with international NGOs throughout the region identified an extensive array of abortion-related research, service provision, commodity distribution, training, and professional and legal advocacy. According to Eva, these activities were less about “promoting abortion” than they were about “reducing unsafe and less safe abortions.” NGOs conducted situational analyses on reproductive health and Knowledge, Attitudes, and Practices (KAP) surveys on abortion and contraception with women and girls. Others have evaluated the availability of MA products in public and private health care facilities. In one country, health workers were trained to monitor maternal deaths, paying special attention to the contribution of unsafe abortion to maternal mortality in their facilities.

In some countries, NGOs have deployed these data in advocacy regarding the harmonization of national laws with the 2003 Maputo Protocol of the African Union, which calls for safe abortion in the case of rape, incest, or when pregnancy threatens a woman’s mental or physical health (African Commission on People and Human Rights, 2003). Some countries have established action plans, with stakeholders in medicine, law enforcement, religion, and other sectors, that endeavor to move toward the integration of the Maputo Protocol. This approach has materialized into legal change in some countries, but not others. Several study participants pointed to the Democratic Republic of Congo (DRC) as an example of an advocacy success story: in March 2018, the government acknowledged that the Maputo Protocol superseded national law (PRB, 2018), and reminded health authorities that safe abortion should be available in public facilities under the conditions specified by the Protocol (SAD, 2019b). In contrast, in Senegal, the abortion law remains unchanged despite advocacy related to the Maputo Protocol (Archer et al., 2018).

Even in countries with legal indications for abortion, study participants explained that provision of and training for MA in the public sector remained limited. PAC remained an important part of their work as women often waited until after they had terminated pregnancy (often unsafely) before seeking care. Some international NGOs were directly involved in “comprehensive” abortion training—safe abortion and PAC—for public sector health workers. Others provided abortion and PAC services in their own clinics (SAD, 2019b). In countries where MA products were registered with national pharmaceutical regulatory agencies, they trained public and private health workers to use them. Additionally, they worked with national partners to include MA in national conversations about reproductive health commodities and supported national health authorities’ efforts to integrate misoprostol into national LEM and protocols for reproductive health care. In several countries, NGOs have conducted “values clarification” workshops to raise awareness of the clinical and legal environment of abortion with a wide variety of stakeholders, including health workers, parliamentarians, journalists, law enforcement officials, youth groups, and professional associations.

While some study participants were directly involved in programming these events, others took more of a behind the scenes approach. For example, Eva differentiated her organization’s “advocacy work” from its “hands-on work.” She understood “hands-on work” as technical in nature: health worker training and registration of commodities. In contrast, she understood her organization’s participation in advocacy as less direct: “We aren’t necessarily the front line to the Parliament or anything like that. I know there are partners who are much more active at that level of Parliamentarians. I would say that we’re not playing that role.” Her organization “supported the movement” by participating in working groups, and providing financial support for national organizations such as the national association of women lawyers, to meet and discuss strategies for legal advocacy.

## “A Product Like Any Other”: Misoprostol Research, Registration, and Marketing

While the previous section describes advocacy, programming, and services that are directly related to abortion, and at times publicly-facing, here I describe how study participants are engaged in strategies with less direct ties to safe abortion, and more oriented toward professions and institutions in private and public sectors of the health system. These strategies entailed conducting research on misoprostol’s safety and efficacy for legal obstetric indications such as PAC and PPH, registering generic brands of misoprostol with national pharmaceutical regulatory agencies, supporting misoprostol’s integration into national LEM and clinical protocols for maternal and reproductive health, and marketing misoprostol directly to health workers and health facilities. In other words, this approach aimed to increase the availability of misoprostol for legal obstetric indications in public and private sectors of the health system, with the knowledge that it would likely be used off-label for abortion.

Some of the earliest activities related to misoprostol in Francophone Africa have involved investigating its feasibility for managing PAC and PPH. A 2004 study in Burkina Faso confirmed that misoprostol was just as effective in treating incomplete abortion as MVA (Dao et al., 2007). In 2012, *BMC Pregnancy and Childbirth* published results from a multi-country (Senegal, Niger, Nigeria, Mauritania, Burkina Faso, and Mauritania) study showing that misoprostol could be used safely for PAC (Shochet et al., 2012). In Senegal, a study showed that midwives in primary health care facilities were able to effectively administer misoprostol for PAC (Gaye et al., 2014). Another study showed that misoprostol could be used by TBAs to prevent PPH (Diadhiou et al., 2011). More recently, a study found that misoprostol was just as effective as oxytocin delivered via Uniject in preventing PPH at the community level (Diop et al., 2016).

In response to this research, MOH in several countries in the region have placed misoprostol on national LEM for the purposes of PAC and preventing and treating PPH. Senegal, for example, placed misoprostol on the national LEM in 2013. In 2014, the Senegalese National Pharmacy of the Ministry of Health integrated misoprostol into its procurement system (Ndao et al., 2014). Following these developments, at least one generic brand of misoprostol has been registered in each country in the Francophone region (SAD, 2019b). A 2019 report on MA commodities in Africa stated that in Senegal, misoprostol was available in packets of three under the brand name Misoclear for $1.75 (approximately 1000 CFA francs) (Mann Global Health, 2019). Study participants in Senegal reported a slightly higher price of between 2,000 and 3,000 CFA francs. In some countries, there are multiple brands of misoprostol: three in Mali and Benin, and two in Niger (IPPF, 2020). As part of their regional strategy, safe abortion advocates encouraged the presence of multiple brands of misoprostol to keep prices low, thereby increasing access to the drug (SAD, 2019b).

With the exception of Senegal, where abortion is not permitted under any circumstance, there is at least one legal indication for abortion in all of the Francophone countries. In Côte d’Ivoire and Chad, there are five and six indications, respectively. In countries with legal indications for abortion, combination MA packs of mifepristone and misoprostol have been registered (SAD, 2019b). Similarly, the “combi-pack” is registered and available in other African countries with legal indications for abortion, such as Ethiopia, Mozambique, Uganda, and Zambia (Mann Global Health, 2019). John, who worked with an international reproductive health NGO with operations in Senegal and other countries in the region, expressed doubts that combi-packs would be registered in Senegal given its highly restrictive abortion law.

Safe abortion advocates described the pharmaceutical system in Francophone Africa as highly “centralized,” with close ties to French wholesalers (SAD, 2019b). Local commercial distributors may be reluctant to take on “the extremely slow and delicate process of registration” with national pharmaceutical registration authorities, which in some places has taken up to three years (Mann Global Health, 2019). In Senegal, organizations that register drugs with national pharmaceutical regulatory agencies are prohibited from directly engaging in social marketing. Consequently, international NGOs that have registered misoprostol have outsourced social marketing to pharmaceutical wholesalers in the private sector. These commercial distributors procure the drug from a French company, and in turn promote and sell the drug to pharmacies and health facilities (Mann Global Health, 2019). According to Zeinab, who worked with an international reproductive health NGO in Senegal, these companies “created demand” on the part of pharmacists and health workers by providing them with information about how to dispense, prescribe, and use misoprostol for authorized purposes such as PPH and PAC. When confronted with health professionals’ concerns about misoprostol’s abortifacient qualities, she explained that the social marketing agents emphasized the fact that the drug “saves lives,” and that it’s a “product like any other” that “should be accessible.” In other words, misoprostol distributors framed the drug in public health terms to highlight its legitimacy in obstetric care in a restrictive legal context.

For some study participants, it was precisely the privatized nature of misoprostol procurement and distribution, along with national recognition of misoprostol’s legal obstetric indications, that had the potential to revolutionize access to safe abortion. Despite legal indications for abortion in all Francophone countries (except Senegal), MA provision in the public sector remains low (SAD, 2019b). Adja, who worked for an international reproductive health NGO in Senegal, pointed out that in the public system, “there are always stock outs” of medication, meaning that health workers may have difficulty procuring misoprostol even for legal indications of PAC and PPH. Working through the private health sector, where pharmaceutical wholesalers procured supplies and sold them to pharmacies and health facilities, offered a more reliable approach to ensuring access to misoprostol.

Catherine, who worked for a philanthropic organization, described misoprostol as a “game changer” for abortion access in Francophone Africa: “Misoprostol is such a subversive and exciting thing because if we can get this stuff on shelves and get pharmacists who know how to get it out…I mean, work within the provisions…it feels like it could be big.” Catherine discussed how MA had been “revolutionary” in countries like India and in parts of Anglophone West Africa. “We’ve seen some places in Anglophone West Africa where MA has already exploded. Ghana is just off the shelves. A pretty amazing amount of product is sold.” For her, medication abortion was a “story waiting to be told” in Francophone Africa. It was just a matter of “figuring out what—following that story and pushing it where it can be pushed.”

John, who worked for an international reproductive health NGO, echoed Catherine’s vision of MA when he said that revising abortion laws should no longer be the “holy grail” of abortion advocacy. Instead, getting misoprostol directly “into women’s hands” through pharmacies should be the focus. Similarly, Julia, who had worked with an international reproductive health NGO for many years, expressed that working “outside” the government health system, through private pharmacies, was the best way to reduce unsafe abortion in places with under-resourced health systems.

For Jacques, who supervised the regional MA strategy for an international NGO, the privatized approach to registering and distributing misoprostol was a double-edged sword. On the one hand, getting the drug into private pharmacies ensured access to the drug for women who could reach these facilities and purchase the medication. On the other hand, procuring an abortifacient drug within the public health system remained a serious challenge. He characterized the act of registering the drug for PPH as a “big battle” because “everyone knows that it’s a product that is used to make abortions safer.” He joked that even the MOH “hid” behind obstetric indications like PPH and PAC to place misoprostol on the national LEM. For Jacques, this approach made people reluctant to order misoprostol “publicly” in government health facilities, even for legal indications. “Everyone knows that they need the product,” he said, “but no one will say, ‘listen, we need to order miso.’ Jacques described a situation in one Francophone country where a multilateral agency donated a quantity of misoprostol to the MOH, which was “only buying contraceptives.” The MOH then sent the drug to “university hospital centers,” but not secondary health facilities. In other words, in the public sector, misoprostol was not always, as Zeinab had suggested, a “product like any other,” precisely because the medication is negatively associated with clandestine abortion.

## The “Stigma” of Abortion

Although several participants pointed to the 2012 OP as an important catalyst to the MA revolution in the region, abortion has factored into the landscape of global reproductive governance since at least the introduction of PAC across the region starting in the late 1990s. While PAC was recognized in the 1994 ICPD as a harm reduction approach to the public health problem of unsafe abortion, health workers and health authorities in countries with restrictive abortion laws have had to frame PAC, and the MVA syringe in particular, in ways that distinguish the intervention from pregnancy termination to remain in compliance with national prohibitions on abortion and the US GGR (Suh, 2021).

Study participants who worked for philanthropic agencies recognized that safe abortion had long been part of the reproductive health, or more broadly, “population” portfolios of their employers. Roberta alluded to “interest” in intersections between “climate change and environmental issues” and “reproductive health access in the Northwest and along the Sahel.” Maxine described hearing from former colleagues in West Africa “about European concerns about migration and feel that this is playing into—in negative ways—the increased attention to West Africa’s population.” Within her agency, however, Maxine believed that work on environment and reproductive health had traditionally been kept “separate.” Roberta agreed, saying “I think there’s been concern about how you talk about it together and the potential negative incentives of linking them together.” She went on to describe, however, how her agency intended to “test the waters” in supporting a project that brought together reproductive health, agriculture, and conservation in an East African country.

Regardless of where one draws the start line for the MA revolution in Francophone Africa, it is clear that global abortion politics significantly complicate stakeholders’ activities related to misoprostol. The GGR prevents national organizations that receive USAID funding for family planning from using money from other sources to do abortion-related work. Consequently, international NGOs and donor agencies exercise caution in distributing abortion-related work and funding to national organizations. Eva described how her NGO ensured that, in the Francophone countries where they worked, their contracted social marketing agencies did not “touch” anything related to abortion. Her NGO had to “separate out” misoprostol and combi-packs from their national partners’ portfolios.

Maxine described her philanthropic agency as one that “places a lot of emphasis on funding local organizations and not international NGOs.” The GGR affected her organization “in small and large ways.” Sometimes, she had to scrap plans for collaborating with an organization when it was discovered that they’d signed the GGR. In some situations, her organization had to increase funding for grantees that refused to sign the GGR and therefore lost USAID funding. When presented with opportunities for collaborating directly with USAID, her organization had to consider whether USAID’s “bigger dollars and influence” were worth “compromising on the abortion piece.” Sometimes, they decided “to go it alone without their support.” She described the reinstatement of the GGR in 2017 as having “a chilling effect” in terms of planning meetings and conferences at higher levels related to reproductive health:

I was at a meeting the other day that was three quarters contraception, one quarter abortion, and no one from AID could come to the meeting…It’s definitely a problem. There’s plenty of other examples of meetings and conferences where all the abortion talk has to be pushed to one end of the meeting so the USAID folks could leave the room before any discussion of abortion begins.

The 2012 OP has simultaneously served as a catalyst to organizing regionally around safe abortion and heightened political anxieties about abortion. Two of the OP’s donors, USAID and the Gates Foundation, do not fund abortion-related work. Due to the “stigma” of abortion, according to Catherine, “the OP, which is family planning specific, has been very resistant to allowing anything related to abortion to enter the Partnership.” Discussions related to abortion have been “limited” to PAC. Even the Gates Foundation had to be reassured of the programming connections between PAC and family planning (Curtis et al., 2019). While Catherine recognized the challenges presented to abortion stakeholders, she did not believe the OP was fundamentally resistant to abortion. “The OP is a commitment of nine governments to do better with family planning,” she explained. “The needs are immense, and the gains are fragile. They’re not the opposition. The OP is not opposing abortion work: they’re protecting very important political gains they’ve made.” Instead, she believed that MA advocates had to pragmatically “harness” the research, programming, and advocacy synergies generated by the OP in ways that moved the MA agenda forward.

Mariam, who worked for an international NGO, seemed less convinced of this approach. She explained how her organization struggled to participate in OP meetings and to integrate abortion into discussions of reproductive health commodities. “We’ve never been invited to these OP meetings which only discuss contraception.” Her organization’s solution was to continue “pushing” the issue and speaking “openly” about abortion.

National abortion politics further complicated the integration of misoprostol into health systems. Laila, a midwife in Senegal who had previously worked with the MOH and now worked with an international NGO, described how “when they started giving misoprostol to *matrons* (TBAs) for PPH, they made them record the serial number and link to each patient used.” This was a way to “safeguard” an abortifacient drug that “shouldn’t be used like aspirin.” Mariam and Eva, each representing a different international NGO, described a situation in one Francophone country where, shortly after training a group of health workers to use misoprostol, the MOH released an official statement warning health workers to limit misoprostol use and prescription to legal obstetric indications. Mariam described how, in another country, health workers reported that MOH officials had removed misoprostol from their facility because of suspicions that it was being used illegally to provide abortions. “They completely stopped supplying misoprostol,” Mariam explained, “so the health workers had to hide and use their own money to buy misoprostol and sell it to women in the facilities.” She went on to describe how in such a political context, “sometimes you have the impression that you take one step, and then there’s something that pushes you back.” Eva echoed her concerns when she said “it just shows that in the region while things are advancing, there is still a lot of possibility for things to go quickly backwards.”

Other study participants suggested that Francophone Africa was a particularly difficult place to work on MA because of institutionalized abortion stigma, even in countries with legal indications for abortion. Maxine contrasted the region to Ethiopia, “where we talk much more openly, freely with the government and the Ministry about abortion services.” When I asked John what it was like to promote misoprostol in Senegal or other African countries with restrictive abortion laws, he explained that his organization takes a “step back” when it comes to abortion by focusing on the legal obstetric indications such as PPH and PAC. From a sales perspective, he said, his organization’s hands were “clean” even if others were purchasing and using the drug for abortion. Zeinab, who worked for an international NGO in Senegal, described her activities related to misoprostol as “commercialization.” She distinguished these activities from off-label utilization for abortion, which she acknowledged was happening among some health workers. Her job, was to market the drug, and to “keep a low profile” with respect to the country’s abortion debate.

## Access: “There Are Many Things That Still Block Women”

Despite global and national recognition of misoprostol as an essential medication for maternal and reproductive health, study participants who worked for NGOs identified significant barriers to accessing the drug in both public and private health sectors. Mariam, who worked for an NGO with activities in multiple Francophone African countries, explained: “It’s still difficult for women to get misoprostol because of legal restrictions. There are many things that still block women.” Jacques, who worked for the same NGO as Mariam, pointed out that “just because the product is in the country does not mean that it is accessible.” This observation is relevant not only for women seeking the drug in the private sector, but also health workers in government health facilities. Ayo, who worked in Côte d’Ivoire, estimated that beyond large hospitals, less than 10% of public health facilities stocked the medication. In Senegal, a survey of obstetric care conducted between 2015 and 2016 showed that only 3.4% of referral facilities carried misoprostol (MSAS, CEFOREP, and UNFPA, 2017). Laila explained that despite misoprostol’s integration into national norms and protocols, Senegalese midwives were more likely to have received MVA training for PAC, and that misoprostol training for PPH was still lacking.

One of the main barriers to obtaining misoprostol, despite its availability in pharmacies under various brand names, was the need for a prescription for a legal obstetric indication. Even with a prescription, Jacques pointed out that women may experience difficulty procuring the amount needed to terminate pregnancy. Misoclear, for example, is sold in packets with three 200 mcg tablets. To meet the recommended dose for up to 12 weeks of gestation, a woman would have to buccally, sublingually, or vaginally take 800 mcg (four pills) every three hours until expulsion (Ipas, 2020). This would require four packs of Misoclear. Requests for multiple packets of Misoclear at a pharmacy might raise suspicion, and prompt pharmacy workers to question clients about why they were purchasing this quantity (Mann Global Health, 2019). Alternatively, Jacques explained, the woman might visit several pharmacies to purchase one or two packs of Misoclear at each, an equally challenging process requiring additional time, transportation costs, and possibly multiple prescriptions.

Adja, who worked for an NGO in Senegal, believed that women’s access to misoprostol depended less on prescriptions for legal obstetric indications than on health workers’ willingness “to take the product and give it to women.” Women relied on health workers for misoprostol because “it’s health workers who have the power to get the product.” Jacques agreed, saying, “there are nurses and midwives who are known for providing this kind of help. Often, it’s a business. You do it off the records, and that’s that.” Laila, a midwife in Senegal, believed that misoprostol was mostly “controlled by doctors.” Mariam explained that while obtaining misoprostol directly from health workers might increase some women’s access to the drug, dependence on health workers also left women vulnerable to exploitation. Health workers may purchase a supply of misoprostol and “resell” it to women at a higher price. “This is the kind of dysfunction that comes out of all these restrictions,” she said. “They’ll buy it with their own money and then sell it clandestinely in the system.”

Study participants’ perceptions of the informal routes through which women obtain misoprostol resonate with ethnographic literature on abortion practices in SSA. In countries like Burkina Faso, Kenya, and Tanzania, women obtain misoprostol from multiple sources, including friends, relatives, health workers, pharmacists, and informal pharmaceutical vendors. While some health workers administer misoprostol directly, others dispense it to women and instruct them on how to use it at home. Women may obtain misoprostol from brick and mortar pharmacies with or without a prescription. Although many women seek misoprostol from health workers in the private sector, some health workers in government facilities may provide the service themselves, or refer women to a colleague. Women obtain misoprostol not only from skilled health workers, but also TBAs and other paramedical providers (Izugbara et al., 2015; Drabo 2019; Solheim et al., 2020; Ouedraogo and Juma, 2020).

Jacques believed that compared to urban women, rural women had less access to misoprostol and relied on “street” medication (also colloquially described as “Chinese” medicine because such pharmaceuticals may be produced in Asian countries like China or India). Ayo explained that for many women, street medication was much more affordable than misoprostol purchased at a pharmacy or through a health worker. “Instead of paying 5000 CFA francs for misoprostol, she can buy Chinese medicine for between 200 and 500 francs.” Both Jacques and Ayo asserted that while such purchases might be more affordable, women faced the risk of encountering inauthentic drugs or receiving inadequate information related to dosage or gestational age, all of which could lead to complications. “In all of the Francophone African countries, we have these people who make these abortifacients available,” Jacques said. “But what is the quality of that medication? What is the quality of the prescribed dosage? That’s the problem. In these circuits you can often find good miso or bad miso, or drugs that are supposed to be miso but are not.”

Jacques disagreed with people who pointed to the risk of complications as a reason to restrict misoprostol because women were “not ready” for self-medicated abortion. “It’s not true,” he argued. For him, the problem was the gap between pharmaceutical distribution systems and clients:

Our pharmaceutical distribution system has completely missed its target. The environment has evolved, but our pharmacies and our distribution policies have not evolved. We wait for the client to come, thinking it’s the client who has to come to us even if they are 100 or 120 km away.

According to Jacques, this gap between distribution policies and clients’ needs essentially “abandoned” those populations who lived far from pharmacies to street medication.

## Discussion

Although SSA’s maternal mortality ratio has reduced by 38% since 2000 (WHO, 2019d), the pharmaceutical revolution in reproductive health envisioned by advocates of misoprostol has yet to materialize in Francophone Africa. Attention to how misoprostol’s pharmaceutical regime is unfolding in this region reveals a great deal about the opportunities and limitations of pharmaceutical solutions to public health problems. Through its multiple “biographies,” misoprostol has generated partnerships between transnational stakeholders invested in reducing fertility and maternal mortality. At the same time, misoprostol’s abortifacient capacity complicates safe abortion advocates’ relations with NGOs and national health authorities that receive support from donors like USAID. Abortion stigma may delay misoprostol’s integration into national LEM, clinical protocols, and pharmaceutical supply systems. Demand for generic brands of misoprostol in the private sector does not necessarily translate into access for low-income and rural women. As an abortifacient, misoprostol’s connection to neo-Malthusian logics of population control threatens the gains won by FP 2020 in revitalizing global commitments to fertility reduction.

Misoprostol’s legal obstetric indications, PAC and PPH, firmly position the medication in harm reduction approaches to maternal and reproductive health (Erdman, 2011; Hyman et al., 2013; Kulczycki, 2016). This has enabled partnerships with USAID, an enormously influential donor in global maternal and reproductive health that accounted for over two thirds of family planning funding between 2003 and 2013 (Grollman et al., 2018). In Senegal, for example, USAID funded a study in 2009 to test the feasibility of providing misoprostol for PPH at the community level (Ortiz et al., 2010). In 2011, USAID awarded Child Fund, an NGO, a $40 million grant over five years to expand health care provision at the community level, including the provision of misoprostol for PPH by TBAs (Ennulat, 2016). Due to anti-abortion funding policies such as the Helms Amendment and the GGR, USAID neither supports national health authorities in procuring misoprostol supplies, nor does it permit its contracting agencies to purchase the medication. Similarly, USAID supported research, training, and programming related to PAC in Senegal, but would not support the procurement of MVA because it is an abortifacient (Curtis, 2007; Suh, 2021).

In 2017, President Trump extended the GGR to organizations that receive US funding for infectious disease (Singh and Karim, 2017). More than ever, study participants were keenly aware of the need to keep MA activities separate from national partners who received funding from USAID. Some stakeholders, however, have been heartened by the injection of non-US sources of bilateral support for MA (and safe abortion more generally) in the region. Others have called for new forms of partnership that fund MOH directly rather than international NGOs. These kinds of partnerships would strengthen the capacity of national governments to procure and distribute technologies (SAD, 2019b).

Misoprostol stokes enduring tensions related to professional authority over reproduction: who can use the medication, where, and for what purpose. The medication’s safety and effectiveness in preventing PPH and PAC have facilitated its integration into LEM throughout Francophone Africa. Yet, study participants pointed to gaps in health worker training and medication supplies in national health systems in countries where they worked. To a certain extent, these gaps reflect continuing debates about the role of TBAs in obstetric care (MacDonald, 2020). At the same time, the informal dispensing of misoprostol by doctors, nurses, midwives, and pharmacists suggests that formal health workers are actively facilitating safer, albeit illegal, abortions.

Starting with the 1974 Helms Amendment, and followed by the 1984 GGR, anti-abortion advocates have actively shaped the terrain of global reproductive health funding and policy (Crane, 1994; Barot, 2013). Misoprostol may offer a new frontier of scientific intervention for global anti-abortion efforts. By framing misoprostol as a poorly evidenced, sub-standard form of care for PPH, these actors aim to restrict access to abortion. For example, Maternal Life International is a Christian organization that, in addition to providing emergency obstetric care in various African countries (and Haiti), promotes couple-based natural contraception. In a 2011 letter to WHO, the organization explicitly expressed its opposition to the use of misoprostol for abortion, suggested that the “real agenda” of “misoprostol advocates” was “not evidence-based obstetrical care but rather unregulated medical abortion,” and affirmed that ethical medical care necessitates “a proscription against abortion” (WHO, 2011a).

Study participants were eager to harness commonalities across Francophone countries, such as a tightly organized pharmaceutical market and the prominence of Islam (SAD, 2019b), toward a regional approach to expanding access to misoprostol, and safe abortion more broadly. While many were enthusiastic about misoprostol’s potential to transform the landscape of reproductive health care in the region, they also described it as an exceptionally challenging place to do abortion-related work. Some bemoaned the reluctance they perceived on the part of the regional OP and national health authorities to engage with safe abortion. They were careful to keep MA “separate from” any national organizations that received USAID funding. In contrast to countries like Ghana and India, where MA was “flying off the shelves,” they believed the region’s burdensome pharmaceutical registration system, coupled with widespread abortion stigma, hampered procurement and distribution of these drugs.

And yet, some of the abortion stigma framed as exceptional to Francophone Africa appears to be relevant in other countries. Maxine described Ethiopia as a place where the MOH was “open” to engaging with abortion. Since 2005, Ethiopia has permitted abortion in cases of rape, incest, fetal malformation, or if the pregnant woman is under the age of 18. A woman’s “word” is considered sufficient to establish her age or that she experienced rape or incest (Blystad et al., 2019). The national society of obstetricians and gynecologists played a prominent role in advocating for abortion law reform (Holcombe, 2018). Yet, a 2016 study of legal abortion provision at public and non-profit facilities showed that despite a favorable political climate, health workers continue to struggle with adjudicating between legitimate and illegitimate requests for abortion (McLean et al., 2019). John, who worked on MA in Francophone Africa, revealed that in a previous position with an NGO in a southern African country, he had been warned directly by the MOH not to cross the line into legal advocacy for abortion. In India, where medication abortion is permitted in certified health facilities up to seven weeks and misoprostol and mifepristone are widely available at private pharmacies (Boler et al., 2009), an estimated 67% of abortions are classified as unsafe (Yokoe et al., 2019). In Ghana, where abortion is permitted for several indications, a 2016 study in the capital city of Accra showed that only half of pharmacies stocked misoprostol, with pharmacies in less affluent neighborhoods less likely to do so (Ganle et al., 2019).

Despite these inconsistencies, the language of exceptionalism remains a formidable tool in constructing Francophone Africa as a place particularly in need of resources and attention related to population. Critical development scholar Betsy Hartmann argues that the current focus on reducing fertility in SSA hinges on racialized perceptions of African fertility as “distinctive and dangerous” (2014). Population interventions are predicated on the “exceptional” nature of high fertility in SSA, despite a wide variation in fertility rates throughout the continent, and the existence of similar demographic patterns in other parts of the world during historical periods characterized by high rates of poverty and inequality (Hartmann, 2014). The OP, which many safe abortion advocates perceive as a unique opportunity to harness regional synergies, is predicated on Francophone Africa’s exceptional demographic indicators.

FP 2020 and regional offshoots like the OP are the most recent interventions in a longer history of population interventions in SSA. For example, in 1980, Senegal was the first country in SSA to receive a structural adjustment loan from the World Bank and the IMF. Release of the third loan during the late 1980s was conditioned upon the establishment of a concrete population policy with support from USAID, Pathfinder International, and UN Population Fund (UNFPA) (Robinson, 2017). More generally, countries in SSA with high levels of structural adjustment debt are more likely to have articulated population policies (Robinson, 2015).

While Maxine describes population and climate change as a “European concern,” population and development scholars have traced the “securitization” of climate change and population as an American concern since at least the mid to late 2000s (Hartmann, 2014; Sasser, 2018). Scarcity in natural resources like land and water due to climate change fuels conflict, which in turn propels migration from SSA to the global North. In the post-9/11 War on Terror, the region’s emerging identity as a recruitment zone for organizations like Al-Qaeda has provided an additional geopolitical rationale for the expansion of US military intervention into Francophone Africa. None of the study participants viewed misoprostol, or MA more generally, as a tool of population control in Francophone Africa. Instead, they understood misoprostol as an important harm reduction approach to addressing the problem of maternal mortality. Yet, donors in the global North (including USAID) have long cultivated an interest in abortion as a technological strategy in the solution of population problems. If abortion and contraception represent two sides of the same neo-Malthusian coin, misoprostol is flexible enough to achieve both of these “jobs” within overlapping regimes of global reproductive governance.

Along with magic bullet interventions that generate statistical measures of cost-effectiveness, twenty-first century global health approaches have incentivized engagement with the private sector. In Senegal, for example, international NGOs have contracted private logistics operators to supply government health facilities with contraceptives. By closely monitoring the private operators’ performance in delivering and taking inventory of contraceptives, this approach endeavors to prevent contraceptive stock-outs in the public sector (Duclos et al., 2019). Some of my study participants were keen to harness the private sector to increase access to misoprostol throughout the Francophone region. Private organizations, with support from international NGOs, do the work of registering and marketing misoprostol and other MA drugs. They believed the presence of multiple brands on the market generated competition between manufacturers, thereby keeping prices affordable for consumers. Sales of misoprostol provide concrete evidence of demand in the Francophone region. In 2018, DKT International, an international reproductive health NGO, sold over a million packets of misoprostol in Benin, Cameroon, the DRC, Côte d’Ivoire, Mali, and Togo (DKT International, 2018). In this sense, the market is key to getting life-saving drugs “into women’s hands.”

Given the lack of evidence that neoliberal health reform in SSA has improved maternal health outcomes (Thomson et al., 2017; Sommer et al., 2019), it is remarkable that the misoprostol revolution would hinge on its location in the private sector. It should come as no surprise that study participants with direct experience with the public health sector were more critical of a privatized, pharmaceuticalized approach to reproductive health. Laila, Ayo, Jacques, and Mariam, all African nationals, described unequal distributions of reproductive health care between private and public health facilities, wealthy and poor women, and rural and urban women. Privatized distribution of misoprostol reinforced the marginalization of community-based health workers like TBAs upon whom low-income and rural women often depend for maternal and reproductive health care. Although multiple generic brands of misoprostol may be available for purchase in formal and informal sectors of the health system, the drug may be unaffordable for low-income women, or altogether inaccessible for rural women who live far from a pharmacy. Even if a woman is able to purchase misoprostol, she may receive inaccurate information about how and when to use the drug, and what to do if complications arise.

Studies from middle- and low-income countries show that low-income women in particular are more likely to receive inaccurate information about misoprostol or inauthentic medication (Footman et al., 2018). In turn, such women may be more likely to experience complications requiring PAC. Although PAC has been widely accepted as a harm reduction strategy for unsafe abortion, in many developing countries PAC services are limited in quality and availability, especially at lower levels of the health system (Owolabi et al., 2018). In countries like Brazil (Zordo, 2016), El Salvador (Oberman, 2018), Senegal (Suh, 2021), and the United States (Eckholm, 2015), PAC serves as a doorway to criminalization if health workers suspect that a patient has illegally terminated pregnancy. Maternal mortality reduction strategies that prioritize access to drugs like misoprostol may thus exacerbate inequalities in reproductive health outcomes and experiences among the very populations they aim to serve.

As a feminist advocate of reproductive justice, I support access to technologies like misoprostol that *empower* women through granting reproductive autonomy in contexts where legal abortion is highly restricted, and *benefit* them by reducing the risk of obstetric complications and death (Layne et al., 2010). Attempts to restrict misoprostol to “protect” women in SSA from the dangers of sub-standard care outside of hospitals conveniently overlook profound inadequacies in obstetric care in health facilities. As lockdowns and other disruptions related to the global Covid-19 pandemic have threatened access to health care worldwide, it is more important than ever to ensure women’s access to life-saving drugs like misoprostol (Kumar, 2020). At the same time, privatized approaches to reproductive health amplify broader inequalities in access to resources according to age, gender, geography, and race. Neoliberal discourses of empowerment and self-actualization through rational consumption of health services or goods such as family planning or facility-based delivery obscure the gendered and racialized anti-natalism of twenty-first century approaches to maternal and reproductive health (Bhatia et al., 2019; MacDonald, 2019). While the presence of misoprostol in pharmacies and hospitals increases access to safe reproductive health care for those who can afford the drug, it also abandons the most vulnerable women to cope with discriminatory abortion laws and under-resourced public health systems while aspiring to responsible reproductive behavior.

## Data Availability

The raw data supporting the conclusions of this article will be made available by the authors, without undue reservation.
